# Supplementary material for: Reduction in spontaneous and iatrogenic preterm births in twin pregnancies during COVID-19 lockdown in Melbourne, Australia: a multicenter cohort study
Source: BMC Pregnancy Childbirth. 2023 Dec 11;23:846. doi: 10.1186/s12884-023-06137-9 (PMC10712149; doi:10.1186/s12884-023-06137-9)
Supplement: Supplementary file 1 — Additional file 1: Supplementary Table 1. Primary and secondary outcomes for all pregnancies in the pre-pandemic, exposure 1 and 2 cohorts (Unadjusted RR). Supplementary Table 2. Infant outcomes in the pre-pandemic, exposure 1 and exposure 2 cohorts (Unadjusted RR). Supplementary Table 3. Primary and secondary outcomes for all pregnancies in the pre-pandemic, exposure 1 and 2 cohorts (Sensitivity analysis excluding terminations of pregnancies and major birth defects). Supplementary Table 4. Infant outcomes in the pre-pandemic, exposure 1 and exposure 2 cohorts (Sensitivity analysis excluding terminations of pregnancies and major birth defects). Supplementary Table 5. Primary and secondary outcomes for all pregnancies in the pre-pandemic, exposure 1 and 2 cohorts (Sensitivity analysis retaining sex-discordant twins only). Supplementary Table 6. Infant outcomes in the pre-pandemic, exposure 1 and exposure 2 cohorts (Sensitivity analysis retaining sex-discordant twins only). [file 12884_2023_6137_MOESM1_ESM.docx]

**SUPPLEMENTAL FILES**

**Supplementary Table 1:** Primary and secondary outcomes for all pregnancies in the pre-pandemic, exposure 1 and 2 cohorts (Unadjusted RR)

| Outcomes | Pre-pandemic  n=1219 | | Exposure 1  n=433 | | Exposure 2  n=615 | | Exposure 1 RR | | | Exposure 2 RR | | |
| --- | --- | --- | --- | --- | --- | --- | --- | --- | --- | --- | --- | --- |
|  | **n** | **%** | **n** | **%** | **n** | **27%** | **RR** | **95% CI** | **P value** | **RR** | **95% CI** | **P value** |
| Preterm birth < 37 wk | | | | | | | | | | | | |
| Total | 833 | 68.3 | 273 | 63.1 | 438 | 71.2 | 0.95 | 0.90-1.00 | **0.034** | 1.03 | 0.98-1.08 | 0.206 |
| Spontaneous | 247 | 20.3 | 82 | 18.9 | 136 | 22.1 | 0.99 | 0.95-1.03 | 0.616 | 1.02 | 0.98-1.06 | 0.391 |
| Iatrogenic | 586 | 48.1 | 191 | 44.1 | 302 | 49.1 | 0.96 | 0.91-1.01 | 0.117 | 1.01 | 0.96-.06 | 0.630 |
| Preterm birth < 34 wk | | | | | | | | | | | | |
| Total | 280 | 23.0 | 86 | 19.9 | 168 | 27.3 | 0.97 | 0.92-1.01 | 0.147 | 1.04 | 0.99-1.08 | 0.064 |
| Spontaneous | 129 | 10.6 | 45 | 10.4 | 72 | 11.7 | 1.00 | 0.97-1.03 | 0.993 | 1.01 | 0.98-1.04 | 0.449 |
| Iatrogenic | 151 | 12.4 | 41 | 9.5 | 96 | 15.6 | 0.97 | 0.93-1.00 | 0.067 | 1.03 | 0.99-1.06 | 0.100 |
| Preterm birth < 28 wk | | | | | | | | | | | | |
| Total | 58 | 4.8 | 23 | 5.3 | 44 | 7.2 | 1.00 | 0.98-1.03 | 0.880 | 1.02 | 0.99-1.04 | 0.071 |
| Spontaneous | 30 | 2.5 | 12 | 2.8 | 22 | 3.6 | 1.00 | 0.98-1.02 | 0.786 | 1.01 | 0.99-1.03 | 0.161 |
| Iatrogenic | 28 | 2.3 | 11 | 2.5 | 22 | 3.6 | 1.00 | 0.98-1.02 | 0.950 | 1.01 | 0.99-1.03 | 0.264 |
| Preterm birth < 24 wk | | | | | | | | | | | | |
| Total | 20 | 1.6 | 7 | 1.6 | 15 | 2.4 | 1.00 | 0.99-1.01 | 0.992 | 1.01 | 0.99-1.02 | 0.244 |
| Spontaneous | 12 | 1.0 | 6 | 1.4 | 8 | 1.3 | 1.00 | 0.99-1.02 | 0.487 | 1.00 | 0.99-1.01 | 0.562 |
| Iatrogenic | 8 | 0.7 | 1 | 0.2 | 7 | 0.8 | 0.99 | 0.98-1.00 | 0.368 | 1.00 | 0.99-1.01 | 0.253 |
| Iatrogenic birth for suspected fetal compromise | | | | | | | | | | | | |
| Total | 249 | 0.4 | 58 | 13.4 | 122 | 19.8 | 0.93 | 0.89-0.97 | **<0.001** | 0.99 | 0.96-1.03 | 0.718 |
| ≥ 37 wk | 53 | 13.7 | 11 | 6.9 | 27 | 15.3 | 0.94 | 0.88-1.00 | **0.035** | 1.02 | 0.98-1.08 | 0.460 |
| < 37 wk | 196 | 23.5 | 47 | 17.2 | 95 | 21.7 | 0.94 | 0.88-0.99 | **0.020** | 0.98 | 0.93-1.02 | 0.349 |
| Mode of birth | | | | | | | | | | | | |
| Vaginal | 361 | 29.6 | 112 | 25.9 | 180 | 29.3 | 0.96 | 0.92-1.01 | 0.152 | 0.99 | 0.95-1.04 | 0.807 |
| Cesarean section after labor onset | 413 | 33.9 | 145 | 33.5 | 235 | 38.2 | 0.99 | 0.94-1.05 | 0.769 | 1.04 | 1.00-1.09 | 0.076 |
| Cesarean section with no labor | 445 | 36.5 | 176 | 40.6 | 200 | 32.5 | 1.04 | 0.99-1.10 | 0.101 | 0.96 | 0.92-1.01 | 0.126 |
| Pregnancy care indicators | | | | | | | | | | | | |
| First antenatal visit ≤12 wk | 717 | 58.8 | 294 | 67.9 | 426 | 69.3 | 1.09 | 1.04-1.15 | **<0.001** | 1.11 | 1.06-1.16 | **<0.001** |
| Severe PPH > 1000ml | 129 | 10.6 | 40 | 9.2 | 66 | 10.7 | 0.99 | 0.95-1.02 | 0.409 | 1.00 | 0.97-1.03 | 0.981 |

*RR*, unadjusted risk ratio; *CI*, confidence interval; *PPH*, post-partum haemorrhage; wk, weeks.

**Supplementary Table 2**: Infant outcomes in the pre-pandemic, exposure 1 and exposure 2 cohorts (Unadjusted RR)

| Outcomes | Pre-pandemic | | Exposure 1 | | Exposure 2 | | Exposure 1 RR | | | Exposure 2 RR | | |
| --- | --- | --- | --- | --- | --- | --- | --- | --- | --- | --- | --- | --- |
|  | **n=2438** | **58%** | **n=866** | **19%** | **n=1230** | **27%** | **RR** | **95% CI** | **P value** | **RR** | **95%CI** | **P value** |
| All-cause stillbirths* | 38 | 3.1 | 13 | 3.0 | 26 | 4.2 | 1.00 | 0.99-1.01 | 0.754 | 1.00 | 0.99-1.01 | 0.953 |
| All-cause stillbirths | 38 | 1.6 | 13 | 1.5 | 26 | 2.1 | 1.00 | 0.99-1.01 | 0.932 | 1.00 | 1.00-1.01 | 0.842 |
| Small for gestational age | 145 | 6.0 | 49 | 5.7 | 70 | 5.7 | 1.00 | 0.98-1.02 | 0.824 | 0.92 | 0.98-1.01 | **<0.001** |
| SCN admissions | 1058 | 43.4 | 333 | 38.5 | 431 | 35.0 | 0.95 | 0.92-0.99 | **0.009** | **1.05** | 0.89-0.95 | **<0.001** |
| NICU admissions | 478 | 19.6 | 171 | 19.8 | 307 | 25.0 | 1.00 | 0.97-1.03 | 0.833 | 1.03 | 1.02-1.08 | 0.236 |
| 5-minute Apgar < 7 (all gestations) | 174 | 7.2 | 60 | 7.0 | 121 | 10.02 | 1.00 | 0.98-1.02 | 0.773 | 0.99 | 1.01-1.05 | 0.00 |
| Congenital anomalies* | 106 | 4.4 | 40 | 4.6 | 49 4.0 | | 1.00 | 0.98-1.02 | 0.981 | 1.00 | 0.98-1.01 | 0.30 |

** Denominator is all pregnancies*

*RR*, unadjusted risk ratio; *CI,* confidence interval; SCN, special care nursery; NICU, neonatal intensive care unit.

**Supplementary Table 3:** Primary and secondary outcomes for all pregnancies in the pre-pandemic, exposure 1 and 2 cohorts (Sensitivity analysis excluding terminations of pregnancies and major birth defects)

| Outcomes | Pre-pandemic | | Exposure 1 | | Exposure 2 | | Exposure 1 aRR | | | Exposure 2 aRR | | |
| --- | --- | --- | --- | --- | --- | --- | --- | --- | --- | --- | --- | --- |
|  | **n=1120** | **54%** | **n=393** | **19%** | **n=556** | **27%** | **aRR** | **95% CI** | **P value** | **aRR** | **95% CI** | **P value** |
| Preterm birth < 37 wk | | | | | | | | | | | | |
| Total | 751 | 67.1 | 246 | 62.6 | 391 | 70.3 | 0.94 | 0.88-0.99 | 0.04 | 1.05 | 0.98-1.10 | 0.09 |
| Spontaneous | 215 | 19.2 | 74 | 18.8 | 117 | 21.0 | 0.96 | 0.92-1.01 | 0.15 | 1.00 | 0.96-1.05 | 0.98 |
| Iatrogenic | 536 | 47.9 | 172 | 43.8 | 274 | 49.3 | 0.97 | 0.91-1.04 | 0.43 | 1.05 | 0.99-1.11 | 0.12 |
| Preterm birth < 34 wk | | | | | | | | | | | | |
| Total | 224 | 20.0 | 71 | 18.1 | 135 | 24.3 | 0.96 | 0.91-1.01 | 0.14 | 1.06 | 1.01-1.11 | 0.01 |
| Spontaneous | 103 | 9.2 | 37 | 9.4 | 59 | 10.6 | 0.99 | 0.95-1.02 | 0.48 | 1.02 | 0.99-1.05 | 0.22 |
| Iatrogenic | 121 | 10.8 | 34 | 8.7 | 76 | 13.7 | 0.98 | 0.94-1.02 | 0.24 | 1.04 | 1.01-1.07 | 0.04 |
| Preterm birth < 28 wk | | | | | | | | | | | | |
| Total | 29 | 2.6 | 13 | 3.3 | 22 | 4.0 | 1.00 | 0.97-1.03 | 0.96 | 1.01 | 0.99-1.04 | 0.21 |
| Spontaneous | 16 | 1.4 | 6 | 1.5 | 11 | 2.0 | 1.00 | 0.98-1.01 | 0.91 | 1.01 | 1.00-1.03 | 0.07 |
| Iatrogenic | 13 | 1.2 | 7 | 1.8 | 11 | 2.0 | 1.00 | 0.98-1.01 | 0.60 | 1.01 | 0.99-1.02 | 0.31 |
| Iatrogenic birth for suspected fetal compromise | | | | | | | | | | | | |
| Total | 230 | 20.5 | 49 | 12.5 | 110 | 19.8 | 0.93 | 0.88-0.98 | 0.00 | 0.99 | 0.95-1.04 | 0.77 |
| ≥ 37 wk | 50 | 13.6 | 9 | 6.1 | 25 | 15.2 | 0.93 | 0.87-1.00 | 0.05 | 1.02 | 0.95-1.09 | 0.20 |
| < 37 wk | 180 | 24.0 | 40 | 16.3 | 85 | 21.7 | 0.93 | 0.87-1.00 | 0.04 | 0.98 | 0.93-1.04 | 0.71 |
| Mode of birth | | | | | | | | | | | | |
| Vaginal | 327 | 29.2 | 100 | 25.5 | 158 | 28.4 | 0.93 | 0.88-0.98 | 0.01 | 0.96 | 0.91-1.01 | 0.09 |
| Caesarean section after labor onset | 378 | 33.8 | 131 | 33.3 | 208 | 37.4 | 0.97 | 0.91-1.03 | 0.28 | 1.02 | 0.97-1.07 | 0.49 |
| Caesarean section with no labor | 415 | 37.1 | 162 | 41.2 | 190 | 34.2 | 1.11 | 1.05-1.18 | <0.001 | 1.02 | 0.97-1.08 | 0.38 |
| Pregnancy care indicators | | | | | | | | | | | | |
| First antenatal visit ≤ 12 wk | 668 | 59.6 | 268 | 68.2 | 389 | 70.0 | 1.04 | 0.98-1.11 | 0.18 | 1.05 | 0.99-1.10 | 0.23 |
| Severe PPH > 1000ml | 124 | 11.1 | 36 | 9.2 | 57 | 10.3 | 0.95 | 0.91-0.99 | 0.01 | 0.97 | 0.94-1.00 | 0.62 |

*aRR*, adjusted risk ratio; *CI*, confidence interval; *PPH*, post-partum haemorrhage; *wk*, weeks.

^a^Adjusted for covariates of preterm birth including maternal age group, first measured BMI, region of birth, SEIFA, interpreter requirement, parity, smoking status and pertussis vaccination using multivariate log-binomial regression models. The log-binomial regression models used the ‘multiple imputations by chained equation’ (MICE) to account for missing data: missing or implausible for weight=85 (3.76%); missing or implausible height = 15 (0.66%), smoking status = 0 (0%), need for interpreter variable=0 (0%), maternal age=6 (0.13%), BMI = 169 (7.34%), region of birth = 11 (0.49%), SEIFA = 35 (1.55%), sex=1 (0.04%).

**Supplementary Table 4:** Infant outcomes in the pre-pandemic, exposure 1 and exposure 2 cohorts (Sensitivity analysis excluding terminations of pregnancies and major birth defects)

| Outcomes | Pre-pandemic | | Exposure 1 | | Exposure 2 | | Exposure 1 aRR | | | Exposure 2 aRR | | |
| --- | --- | --- | --- | --- | --- | --- | --- | --- | --- | --- | --- | --- |
|  | **n=2438** | **58%** | **n=866** | **19%** | **n=1230** | **27%** | **aRR** | **95%CI** | **P value** | **aRR** | **95%CI** | **P value** |
| Adjusted stillbirths | 21 | 0.9 | 9 | 1.2 | 9 | 0.8 | 1.00 | 0.99-1.01 | 0.64 | 0.99 | 0.99-1.00 | 0.15 |
| Fetal growth restriction | 133 | 5.9 | 43 | 5.5 | 59 | 5.3 | 1.00 | 0.98-1.02 | 0.86 | 0.99 | 0.98-1.01 | 0.58 |
| SCN admissions | 998 | 44.6 | 316 | 40.2 | 415 | 37.3 | 0.91 | 0.87-0.95 | <0.001 | 0.87 | 0.84-0.91 | 0.00 |
| NICU admissions | 407 | 18.2 | 148 | 18.8 | 259 | 23.3 | 1.00 | 0.96-1.04 | 0.99 | 1.08 | 1.04-1.11 | 0.00 |
| 5-minute Apgar <7 (term infants) | 26 | 3.5 | 14 | 4.8 | 15 | 4.6 | 0.99 | 0.97-1.02 | 0.72 | 1.00 | 0.97-1.03 | 0.89 |

*aRR*, adjusted risk ratio; *CI,* confidence interval; SCN, special care nursery; NICU, neonatal intensive care unit.

**Supplementary Table 5:** Primary and secondary outcomes for all pregnancies in the pre-pandemic, exposure 1 and 2 cohorts (Sensitivity analysis retaining sex-discordant twins only)

| **Outcomes** | **Pre-pandemic** | | **Exposure 1** | | **Exposure 2** | | **Exposure 1 aRR** | | | **Exposure 2 aRR** | | |
| --- | --- | --- | --- | --- | --- | --- | --- | --- | --- | --- | --- | --- |
|  | **n=390** | **54%** | **n=131** | **19%** | **n=167** | **27%** | **aRR** | **95%CI** | **P value** | **aRR** | **95%CI** | **P value** |
| **Preterm birth <37 wk** | | | | | | | | | | | | |
| Total | 226 | 58.0 | 66 | 50.4 | 98 | 58.7 | 0.89 | 0.80-0.99 | 0.043 | 1.00 | 0.91-1.11 | 0.967 |
| Spontaneous | 81 | 20.8 | 25 | 19.1 | 44 | 26.4 | 0.95 | 0.87-1.04 | 0.283 | 1.03 | 0.95-1.12 | 0.481 |
| Iatrogenic | 145 | 37.2 | 41 | 31.3 | 54 | 32.3 | 0.94 | 0.85-1.04 | 0.242 | 0.97 | 0.88-1.07 | 0.572 |
| **Preterm birth <34 wk** | | | | | | | | | | | | |
| Total | 75 | 19.2 | 18 | 13.7 | 35 | 21.0 | 0.90 | 0.83-0.98 | 0.015 | 1.01 | 0.93-1.09 | 0.852 |
| Spontaneous | 43 | 11.0 | 11 | 8.4 | 23 | 13.8 | 0.94 | 0.88-1.01 | 0.082 | 1.03 | 0.97-1.10 | 0.325 |
| Iatrogenic | 32 | 8.2 | 7 | 5.3 | 12 | 7.2 | 0.96 | 0.91-1.02 | 0.194 | 0.98 | 0.93-1.04 | 0.507 |
| **Preterm birth <28 wk** | | | | | | | | | | | | |
| Total | 19 | 4.9 | 6 | 4.6 | 10 | 6.0 | 0.97 | 0.92-1.01 | 0.138 | 1.00 | 0.96-1.04 | 0.903 |
| Spontaneous | 14 | 3.6 | 4 | 3.1 | 6 | 3.6 | 0.98 | 0.94-1.01 | 0.230 | 0.99 | 0.96-1.03 | 0.659 |
| Iatrogenic | 5 | 1.3 | 2 | 1.5 | 4 | 2.4 | 0.99 | 0.96-1.02 | 0.445 | 1.02 | 0.98-1.03 | 0.573 |
| **Iatrogenic birth for fetal compromise** | | | | | | | | | | | | |
| Total | 74 | 19.0 | 14 | 10.7 | 24 | 14.4 | 0.93 | 0.86-1.01 | 0.070 | 0.96 | 0.89-1.03 | 0.288 |
| ≥37 Weeks | 26 | 15.9 | 5 | 7.7 | 9 | 13.0 | 0.9 | 0.80-1.00 | 0.055 | 0.96 | 0.86-1.07 | 0.499 |
| <37 Weeks | 48 | 21.2 | 9 | 13.6 | 15 | 15.3 | 0.96 | 0.85-1.08 | 0.478 | 0.96 | 0.86-1.06 | 0.429 |
| **Mode of birth** | | | | | | | | | | | | |
| Vaginal | 112 | 28.7 | 34 | 26.0 | 53 | 31.7 | 0.93 | 0.85-1.03 | 0.157 | 0.97 | 0.89-1.06 | 0.496 |
| Cesarean section after labor onset | 126 | 32.3 | 46 | 35.1 | 59 | 35.3 | 1.00 | 0.90-1.11 | 0.968 | 1.04 | 0.95-1.14 | 0.402 |
| Ceasarean section with no labor | 152 | 39.0 | 51 | 38.9 | 55 | 32.9 | 1.07 | 0.97-1.19 | 0.181 | 0.99 | 0.90-1.09 | 0.842 |
| **Pregnancy Care Indicators** | | | | | | | | | | | | |
| First antenatal visit ≤12 weeks | 226 | 58.0 | 90 | 68.7 | 115 | 68.9 | 1.14 | 1.03-1.26 | 0.014 | 1.12 | 1.02-1.23 | 0.016 |
| Severe PPH > 1000ml | 48 | 12.3 | 15 | 11.5 | 17 | 10.2 | 0.98 | 0.91-1.04 | 0.476 | 0.95 | 0.89-1.01 | 0.130 |

*aRR,* adjusted risk ratio; *CI*, confidence interval; *PPH*, post-partum haemorrhage; *wk*, weeks.

^q^Adjusted for covariates of preterm birth including maternal age group, first measured BMI, region of birth, SEIFA, interpreter requirement, parity, smoking status and pertussis vaccination using multivariate log-binomial regression models. The log-binomial regression models used the ‘multiple imputations by chained equation’ (MICE) to account for missing data: missing or implausible for weight = 85 (3.76%); missing or implausible height = 15 (0.66%), smoking status = 0(0%), need for interpreter variable = 0 (0%), maternal age = 6 (0.13%), BMI = 169 (7.34%), region of birth = 11 (0.49%), SEIFA = 35 (1.55%), sex = 1 (0.04%).

**Supplementary Table 6:** Infant outcomes in the pre-pandemic, exposure 1 and exposure 2 cohorts (Sensitivity analysis retaining sex-discordant twins only)

| **Outcomes** | **Pre-pandemic** | | **Exposure 1** | | **Exposure 2** | | **Exposure 1 aRR** | | | **Exposure 2 aRR** | | |
| --- | --- | --- | --- | --- | --- | --- | --- | --- | --- | --- | --- | --- |
|  | **n=780** | **57%** | **n=262** | **19%** | **n=334** | **24%** | **aRR** | **95%CI** | **P value** | **aRR** | **95%CI** | **P value** |
| All-cause stillbirth | 13 | 1.67 | 2 | 0.76 | 2 | 0.6 | 0.99 | 0.97-1.00 | 0.115 | 0.99 | 0.97-1.00 | 0.06 |
| Fetal growth restriction | 145 | 5.95 | 49 | 5.7 | 70 | 5.7 | 1.00 | 0.98-1.02 | 0.848 | 1.00 | 0.98-1.02 | 0.930 |
| SCN admissions | 328 | 42.11 | 97 | 37.02 | 123 | 36.83 | 0.92 | 0.86-0.99 | 0.035 | 0.92 | 0.86-0.99 | 0.03 |
| NICU admissions | 121 | 15.53 | 32 | 12.21 | 69 | 20.66 | 0.93 | 0.88-0.98 | 0.008 | 1.02 | 0.97-1.08 | 0.37 |
| 5-minute Apgar <7 (all gestations) | 60 | 7.72 | 13 | 4.96 | 23 | 6.97 | 0.97 | 0.93-1.01 | 0.113 | 0.99 | 0.95-1.02 | 0.53 |

*RR*, adjusted risk ratio; *CI,* confidence interval; SCN, special care nursery; NICU, neonatal intensive care unit.

^a^Adjusted for covariates of preterm birth including maternal age group, first measured BMI, region of birth, SEIFA, interpreter requirement, parity, smoking status and pertussis vaccination using multivariate log-binomial regression models. The log-binomial regression models used the ‘multiple imputations by chained equation’ (MICE) to account for missing data: missing or implausible for weight = 85 (3.76%); missing or implausible height = 15 (0.66%), smoking status = 0 (0%), need for interpreter variable = 0 (0%), maternal age = 6 (0.13%), BMI=169 (7.34%), region of birth =11 (0.49%), SEIFA = 35 (1.55%), sex = 1 (0.04%).
